# Plants exposed to titanium dioxide nanoparticles acquired contrasting photosynthetic and morphological strategies depending on the growing light intensity: a case study in radish

**DOI:** 10.1038/s41598-023-32466-y

**Published:** 2023-04-11

**Authors:** Akram Vatankhah, Sasan Aliniaeifard, Moein Moosavi-Nezhad, Sahar Abdi, Zakieh Mokhtarpour, Saeed Reezi, Georgios Tsaniklidis, Dimitrios Fanourakis

**Affiliations:** 1 Photosynthesis Laboratory, Department of Horticulture, Aburaihan Campus, University of Tehran, P.O. Box 33916-53755, Tehran, Iran; 2 Department of Horticulture, Faculty of Agriculture, University of Shahrekord, Shahrekord, Iran; 3 Department of Horticultural Sciences, North Carolina State University, Raleigh, NC 27695 USA; 4 Laboratory of Vegetable Crops, Institute of Olive Tree, Subtropical Plants and Viticulture, Hellenic Agricultural Organization ‘ELGO DIMITRA’, 73100 Chania, Greece; 5 Laboratory of Quality and Safety of Agricultural Products, Landscape and Environment, Department of Agriculture, School of Agricultural Sciences, Hellenic Mediterranean University, Estavromenos, 71004 Heraklion, Greece

**Keywords:** Light responses, Photosynthesis

## Abstract

Due to the photocatalytic property of titanium dioxide (TiO_2_), its application may be dependent on the growing light environment. In this study, radish plants were cultivated under four light intensities (75, 150, 300, and 600 μmol m^−2^ s^−1^ photosynthetic photon flux density, PPFD), and were weekly sprayed (three times in total) with TiO_2_ nanoparticles at different concentrations (0, 50, and 100 μmol L^−1^). Based on the obtained results, plants used two contrasting strategies depending on the growing PPFD. In the first strategy, as a result of exposure to high PPFD, plants limited their leaf area and send the biomass towards the underground parts to limit light-absorbing surface area, which was confirmed by thicker leaves (lower specific leaf area). TiO_2_ further improved the allocation of biomass to the underground parts when plants were exposed to higher PPFDs. In the second strategy, plants dissipated the absorbed light energy into the heat (NPQ) to protect the photosynthetic apparatus from high energy input due to carbohydrate and carotenoid accumulation as a result of exposure to higher PPFDs or TiO_2_ concentrations. TiO_2_ nanoparticle application up-regulated photosynthetic functionality under low, while down-regulated it under high PPFD. The best light use efficiency was noted at 300 m^−2^ s^−1^ PPFD, while TiO_2_ nanoparticle spray stimulated light use efficiency at 75 m^−2^ s^−1^ PPFD. In conclusion, TiO_2_ nanoparticle spray promotes plant growth and productivity, and this response is magnified as cultivation light intensity becomes limited.

## Introduction

Global food demand is steadily rising as a result of a growing population^1^. To ensure global food security, further improvements in crop yields are necessary^1,2^. Intensifying agricultural production by increasing the associated inputs is commonly employed as an effective strategy to stimulate plant growth and performance^3–6^. Yet, improving input use efficiency stands out as a more attractive alternative^6^, since the intensification-associated environmental pressure is evaded^7^.

Noteworthy, the application of nanoparticles has proven to be a powerful tool in advancing yield^8^. For instance, the application of titanium dioxide (TiO_2_) nanoparticles has been associated with stimulation of carbohydrate production, chlorophyll formation, as well as improvements in photosynthetic rate and eventually yield^9,10^. These promotive effects are concentration dependent, and vary excessively among species^9,11^. In addition, the positive effect of TiO_2_ nanoparticles on plant growth and productivity depends on the environmental conditions during cultivation^10,11^. In this regard, their application may be exercised when the remaining conditions allow optimal impact on plants. For instance, plant growth and productivity also strongly depends on light availability^12–15^. Light is the main energy source-driver of photosynthesis. Light intensity is the pivotal determinant of plant growth and performance^16–18^. Production of crops in greenhouses and controlled environments has nowadays attracted a lot of attention^19^. There is a need to optimize the photosynthetic photon flux density (PPFD) needed for plant growth in an approach with the highest resource use efficiency^6,18^. TiO_2_ nanoparticles are used to improvemeyield during winter cultivation of plants^20^. However, the effect of either TiO_2_ nanoparticles application or light level has been limitedly addressed. It, therefore, remains unknown whether or not these two factors interact to determine plant yield.

Chlorophyll fluorescence is often employed for non-invasive assessments of the electron transport system properties^21–24^. Polyphasic chlorophyll fluorescence induction curves may be additionally utilized to study the fate of absorbed light energy and provide further insights into the structure and function of the photosynthetic system^25–27^.

TiO_2_ nanoparticles have special properties such as high specific area, easy operation, high absorption capacity, and photocatalytic activity. They have emerged as antimicrobial agents and growth promoters. Chahardoli et al.^28^, showed that promoting effects of TiO_2_ application on the growth of plants are concentration-dependent. Indeed, in low concentrations (50 and 100 mg L^−1^) TiO_2_ nanoparticles had a stimulating effect; while increasing the concentration to 2500 mg L^−1^ impose an inhibitory effect on plant growth^24^. It has been reported that TiO_2_ nanoparticles increase the rate of photosynthesis and plant immunity, leading to a 30% increase in yield^11^. In the study of Hossein et al.^29^, foliar application of 2.5 mg L^−1^ Ti, chlorophyll pigments (chlorophyll a, b, carotenoid), plant biomass, photochemical efficiency of photosystem II (F_v_/F_m_) and electron transfer rate (ETR) of soybean improve under natural light conditions. Choi et al.^30^ reported that TiO_2_ foliar application affected CO_2_ absorption and chlorophyll fluorescence of cherry tomato plants. Foliar spraying of TiO_2_ during cloudy days with low light intensity increased the electron transfer rate from Q_A_ to Q_B_ in the reaction center of photosystem II (ET_0_/RC) and carbon dioxide (CO_2_) stabilization. The use of TiO_2_ nanoparticles for strawberry cultivation in the winter, which is characterized by low light intensity, increased the chlorophyll content, fruit yield, and firmness of strawberry fruit^20^.

Controlled environment agriculture (CEA) has emerged as a new sustainable approach to increase the productivity of crops irrespective of natural environmental variations^27^. However, increasing the efficacy of artificial light in CEA is of vital importance to make it energy-cost effective^31^. There is scarce information on the interaction of light intensity and TiO_2_ nanoparticles in the CEA. Considering that TiO_2_ nanoparticles have photocatalytic activity, they can be capable of increasing the efficiency of light use^32^. Therefore, detailed information on their practical impacts on photosynthesis and crop growth is needed.

The aim of this study was to investigate for the first time the joint inputs of cultivation light level and TiO_2_ nanoparticles application on determining photosynthetic efficiency, biomass allocation and yield in controlled-environment agriculture. Radish (*Raphanus raphanistrum* L.) was employed as a model species, owing to the high harvest index (i.e., edible leaves and tuber), and short growth cycle^33^. The obtained data serve as a conceptual basis for understanding the approaches that plants utilized under different scenarios of light energy inputs, as well as for the stimulation of photosynthetic capacity with the aim of maximizing plant growth and productivity, especially in indoor environments where light use efficiency is of obvious concern.

## Results

### Characterization of TiO_2_

**Figure 1:**
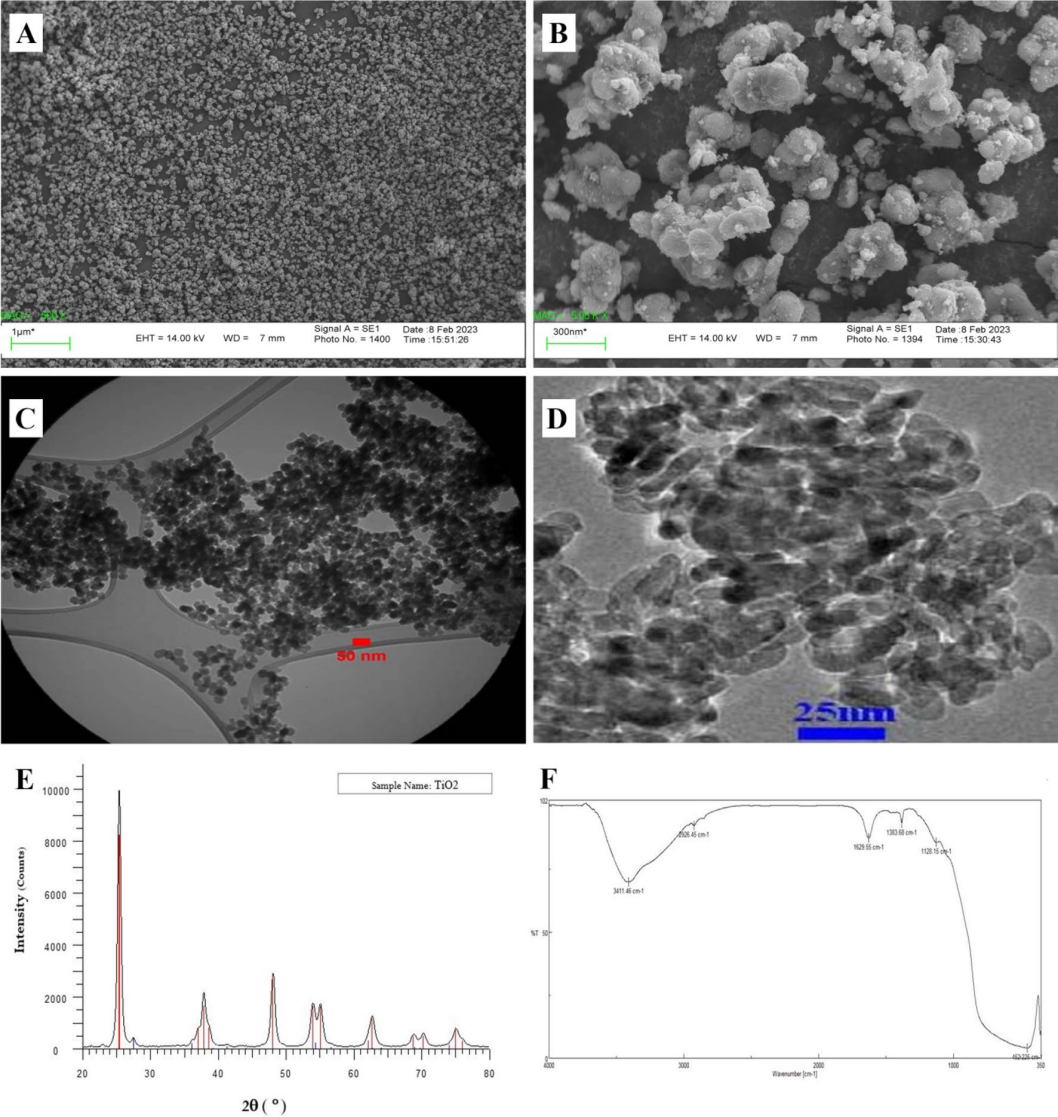
Representative scanning electron microscope (SEM), transmission electron microscopy (TEM) images (**A**–**D**), X-ray diffraction (XRD) pattern (**E**), and fourier-transform infrared spectroscopy (**F**) of titanium dioxide (TiO_2_) nanoparticles employed in the present study.

The surface morphology of TiO_2_ nanoparticles was determined by scanning electron microscope (SEM) and transmission electron microscopy (TEM) (Fig. 1A–D). Briefly, TiO_2_ nanoparticles were spherical or rod-shaped with -32 mV zeta potential and good dispersion. The size of most nanoparticles was in the range of 10–25 (average 18 nm) nm. Using BET method, SSA, pore volume, and average pore diameter were evaluated as 69.679 m^2^g^−1^, 0.2588 cm^3^g^−1^, and 14.857 nm, respectively.

X-ray diffraction showed all diffraction peaks of the anatase phase well (Fig. 1E). The characteristic peak of the anatase phase was 2Ɵ = 25.3^34^. Several sharp peaks are observed in the 2Ɵ range at 20–80°. The sharp peaks in the 2Ɵ range were 25.12°, 38.25°, 48.10°, 54°, 55.1°, 63°, 69.0°, 70.50°, and 75.10°.

The FTIR spectrum of TiO_2_ nanoparticles is shown in Fig. 1F. The used spectrometer has measured vibrations in the wavelength range of 350–4000 cm^−1^. The broad peak at 3411.46 cm^−1^ was related to the intermolecular interaction of the hydroxyl group (‒OH) attached to Ti. The 1629.55 peak corresponds to the -OH bending vibration. The broad peak at 452.225 cm^−1^ is assigned to the bending vibration bonds (Ti–O–Ti) in the TiO_2_ lattice^34^. The observation of the mentioned peaks in the FTIR spectrum indicates the presence of Ti in the nanoparticle structure and the formation of TiO_2_ nanoparticles.

In present study, TiO_2_ foliar application affected chlorophyll fluorescence parameters of radish plants under different light intensities. Our results showed that foliar spraying of TiO_2_ nanoparticles reduced the negative influence of low light intensity on radish. TiO_2_ treatment led to an increase in EI_0_/RC under low light conditions, which indicates that TiO_2_ improves the rate of electron transfer flux in photosystem II reaction centers. In addition, in the reaction center of TiO_2_-treated plants, the dissipated energy flux DI_0_/RC decreased, indicating that TiO_2_ contributed to the photosynthetic light response.

### Increasing cultivation light intensity improved plant growth, and triggered biomass allocation towards the tuber in a TiO_2_ concentration-dependent manner

Plants were cultivated under four light intensities (75, 150, 300 and 600 μmol m^−2^ s^−1^ PPFD), and weekly sprayed with three TiO_2_ nanoparticle levels (0, 50, and 100 μmol L^−1^).

**Figure 2:**
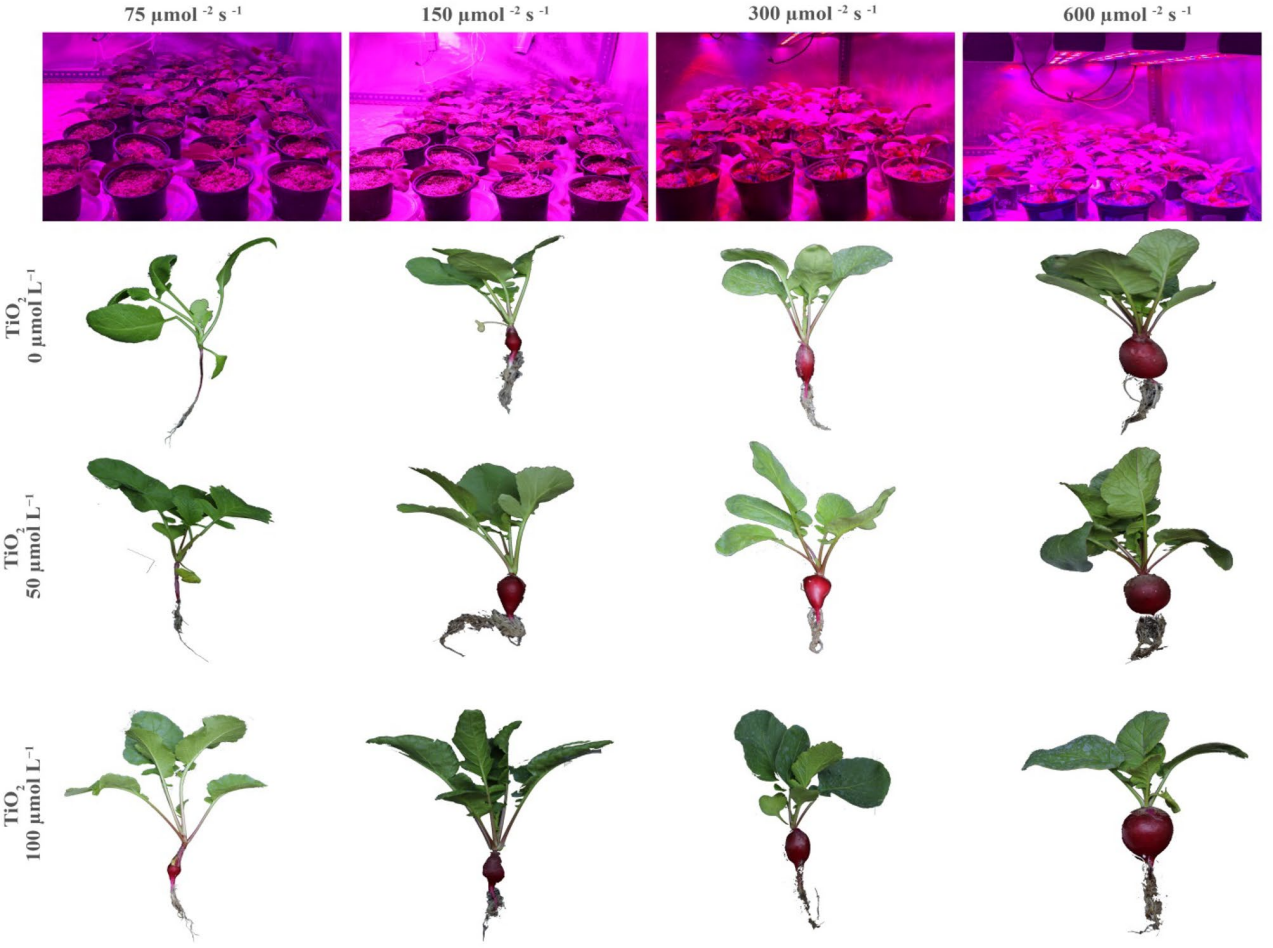
The growth environment (top panels), and representative images of radish cv. Cherry belle plants cultivated under different light intensities and sprayed (once a week, and three times in total) with titanium dioxide (TiO_2_) nanoparticles at different levels.

An increase in cultivation light intensity from 75 to 150 μmol m^−2^ s^−1^ PPFD resulted in a two-fold plant leaf area (Table 1; see also Fig. 2). This increase was mostly due to larger individual leaf area, and secondarily due to an increase in leaf number. A two-step increase in cultivation light intensity from 150 (though 300) to 600 μmol m^−2^ s^−1^ PPFD led to a smaller increase in plant leaf area. This increase was exclusively related to an increase in leaf number.

**Table 1:** Effect of cultivation light intensity and spray treatment (once a week, and three times in total) with titanium dioxide (TiO_2_) nanoparticles on growth and morphology of radish cv.

Light intensity (µmol m^−2^ s^−1^)	TiO_2_ (µmol L^−1^)	Leaf			Petiole length (leaf ^−1^)	Dry weight (g)			Tuber volume (mL)	SLA (cm^2^ g^−1^)	LMR (g g^−1^)	TMR (g g^−1^)
		Number (plant^−1^)	Area (cm^2^ leaf^−1^)	Area (cm^2^ plant^−1^)		Shoot	Tuber	Plant				
75	0	4.0^d^	11.0^d^	43.9^f^	7.11^a^	0.09^e^	0.07^f^	0.15^f^	1.50^d^	520^a^	0.56^ab^	0.44^de^
	50	4.0^d^	17.9^abc^	71.5^e^	5.47^bcd^	0.16^d^	0.11^f^	0.27^ef^	1.00^d^	447^ab^	0.60^a^	0.40^e^
	100	4.0^d^	16.8^c^	67.3^e^	5.88^bc^	0.17^d^	0.13^f^	0.30^ef^	1.00^d^	403^b^	0.56^ab^	0.44^de^
150	0	4.7^bcd^	18.3^abc^	85.2^cd^	6.37^ab^	0.22^cd^	0.21^ef^	0.43^e^	2.00^d^	415^b^	0.50^bc^	0.50^cd^
	50	5.3^ab^	20.8^abc^	109.8^a^	4.93^cde^	0.42^b^	0.37^de^	0.79^d^	9.50^c^	262^c^	0.53^ab^	0.47^de^
	100	4.3^cd^	21.9^ab^	93.4^bc^	7.79^a^	0.25^c^	0.57^d^	0.81^d^	2.50^d^	383^b^	0.30^d^	0.70^b^
300	0	5.7^a^	16.7^c^	94.0^bc^	4.91^cde^	0.43^b^	0.56^d^	0.99^d^	2.00^d^	223^cde^	0.43^c^	0.57^c^
	50	5.0^abc^	21.9^ab^	110^a^	3.92^ef^	0.43^b^	0.89^c^	1.31^c^	9.50^c^	258^cd^	0.33^d^	0.67^b^
	100	5.0^abc^	22.6^a^	110^a^	3.77^ef^	0.41^b^	1.11^b^	1.52^c^	8.50^c^	271^c^	0.27^de^	0.73^ab^
600	0	5.7^a^	17.8^bc^	100^ab^	4.39^def^	0.55^a^	1.32^b^	1.86^b^	19.0^b^	186^de^	0.30^d^	0.70^b^
	50	5.3^ab^	19.4^abc^	103^ab^	3.29^gf^	0.57^a^	1.58^a^	2.14^a^	25.0^a^	182^e^	0.27^de^	0.73^ab^
	100	4.3^cd^	19.0^abc^	76.9^de^	2.17^g^	0.44^b^	1.74^a^	2.18^a^	19.0^b^	177^e^	0.20^e^	0.80^a^

Cherry belle plants. Six replicate plants were assessed per treatment. In traits, where the interaction of the two factors (light intensity, TiO_2_ nanoparticle concentration) was significant, different letters indicate significant differences.

*LMR* leaf mass ratio, *SLA* specific leaf area, *TMR* tuber mass ratio.

At 75, 150, and 300 μmol m^−2^ s^−1^ PPFD, TiO_2_ nanoparticle spray stimulated plant leaf area (Table 1; see also Fig. 2). This increase was exclusively related to an increase in individual leaf area. At 150 μmol m^−2^ s^−1^ PPFD, the low TiO_2_ nanoparticle level (50 μmol L^−1^) was optimal for plant leaf area, while at 75 and 300 μmol m^−2^ s^−1^ PPFD no significant difference was noted among the two TiO_2_ nanoparticle levels. At 600 μmol m^−2^ s^−1^ PPFD, the low TiO_2_ nanoparticle level (50 μmol L^−1^) did not affect plant leaf area, while the high TiO_2_ nanoparticle level (100 μmol L^−1^) decreased it. This decrease was exclusively related to a decrease in leaf number.

Increasing cultivation light intensity resulted in shorter leaf petioles (Table 1). Within each light intensity, TiO_2_ nanoparticle spray decreased leaf petiole in a concentration-dependent manner in all cases, besides one (150 μmol m^−2^ s^−1^ PPFD, 100 μmol L^−1^).

Increasing cultivation light intensity strongly stimulated plant dry weight (Table 1; see also Fig. 2). For instance, plant dry weight was 0.15 g at 75 μmol m^−2^ s^−1^ PPFD, while it was 1.86 g at 600 μmol m^−2^ s^−1^ PPFD. This increase in plant dry weight was mediated via respective increases in both shoot and tuber dry weights.

At each cultivation light intensity, TiO_2_ nanoparticle spray stimulated plant dry weight (Table 1; see also Fig. 2). No significant difference was noted between the two TiO_2_ nanoparticle levels.

Increasing cultivation light intensity generally led to thinner leaves (i.e., lower SLA; Table 1). At 75 μmol m^−2^ s^−1^ PPFD, TiO_2_ nanoparticle spray was also associated with thinner leaves (Table 1). At 300 μmol m^−2^ s^−1^ PPFD, the opposite trend was noted (Table 1).

**Figure 3:**
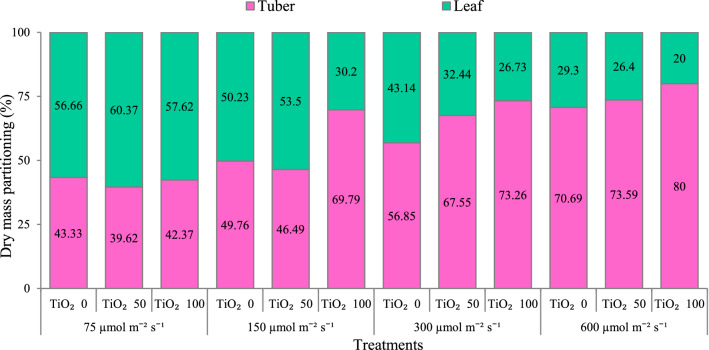
Effect of cultivation light intensity (75, 150, 300 and 600 µmol m^−2^ s^−1^) and spray treatment (once a week, and three times in total) with titanium dioxide (TiO_2_) nanoparticles (0, 50 and 100 µmol L^−1^) on biomass partitioning of radish cv. Cherry belle plants. Six replicate plants were assessed per treatment.

Increasing cultivation light intensity generally decreased dry mass allocation to leaves, and increased dry mass allocation to the tuber (Table 1; Fig. 3). At 300 and 600 μmol m^−2^ s^−1^ PPFD, this trend was magnified by TiO_2_ nanoparticle spray in a TiO_2_ concentration-dependent manner (Table 1; Fig. 3).

### Increasing cultivation light intensity generally improved leaf chlorophyll and carotenoid contents in a TiO_2_ concentration-dependent manner

**Figure 4:**
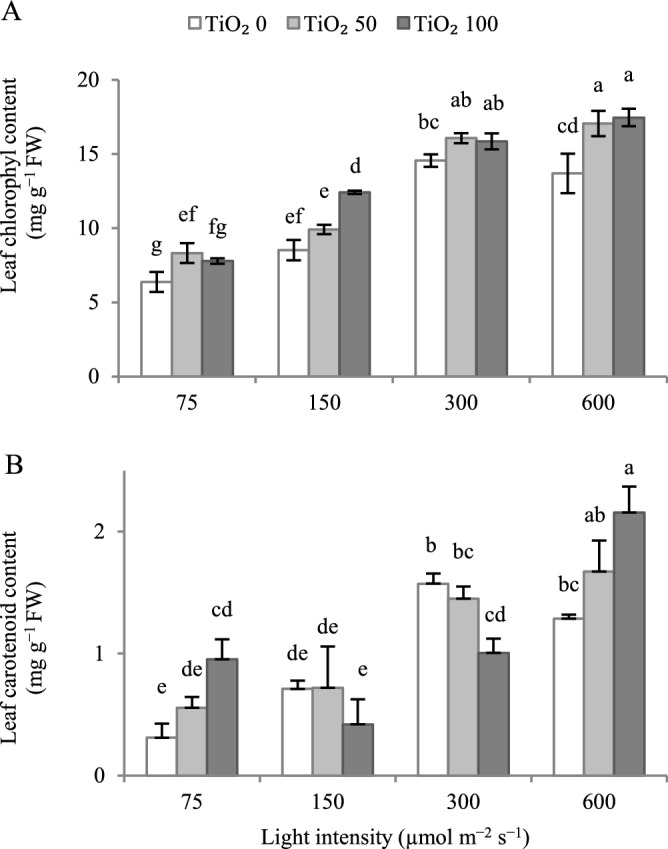
Effect of cultivation light intensity (75, 150, 300 and 600 µmol m^−2^ s^−1^) and spray treatment (once a week, and three times in total) with titanium dioxide (TiO_2_) nanoparticles (open, grey, and dark grey for 0, 50 and 100 µmol L^−1^, respectively) on leaf chlorophyll, and carotenoid contents of radish cv. Cherry belle plants. Six replicate plants were assessed per treatment. Bars represent SEM. In traits, where the interaction of the two factors (light intensity, TiO_2_ nanoparticle level) was significant, different letters indicate significant differences. FW, fresh weight.

Increasing cultivation light intensity up to 300 μmol m^−2^ s^−1^ PPFD led to an increase in leaf chlorophyll and carotenoid contents, while a further increase to 600 μmol m^−2^ s^−1^ PPFD resulted in a respective decrease (Fig. 4).

TiO_2_ nanoparticle spray generally stimulated leaf chlorophyll content, and counteracted the noted decrease at 600 μmol m^−2^ s^−1^ PPFD (Fig. 4A).

At 75, and 600 µmol m^−2^ s^−1^ PPFD, TiO_2_ nanoparticle spray stimulated leaf carotenoid content, and counteracted the noted decrease at 600 μmol m^−2^ s^−1^ PPFD (Fig. 4B). At 150, and 300 µmol m^−2^ s^−1^ PPFD, the high TiO_2_ nanoparticle level (100 μmol L^−1^) tended to decrease leaf carotenoid content, with this effect being only significant at 300 µmol m^−2^ s^−1^ PPFD.

### Increasing cultivation light intensity and applying TiO_2_ nanoparticles generally induce accumulation of soluble carbohydrates in both leaves and tuber

**Figure 5:**
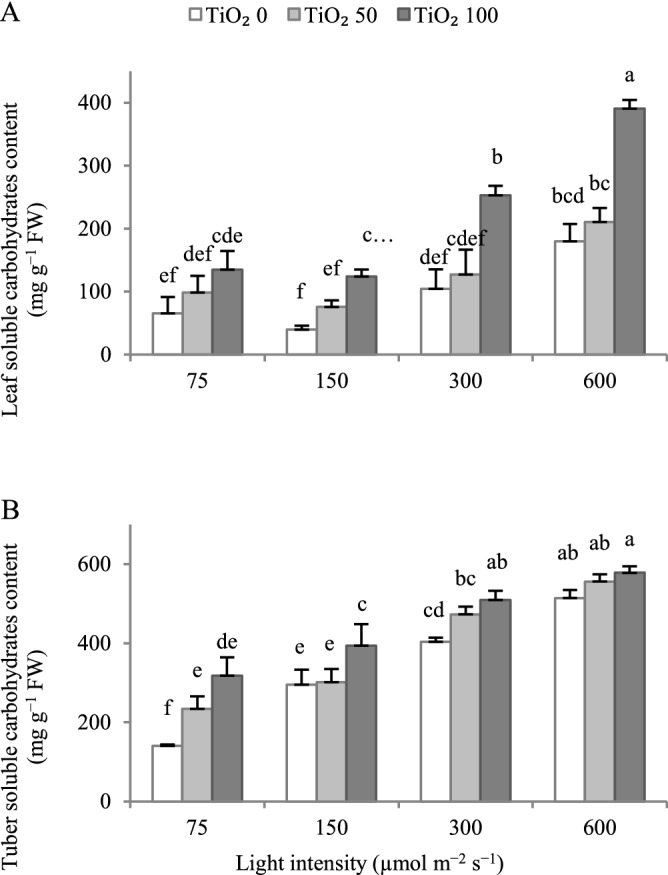
Effect of cultivation light intensity (75, 150, 300 and 600 µmol m^−2^ s^−1^) and spray treatment (once a week, and three times in total) with titanium dioxide (TiO_2_) nanoparticles (open, grey, and dark grey for 0, 50 and 100 µmol L^−1^, respectively) on total carbohydrates content in leaves and tuber (**A**,**B**, respectively) of radish cv. Cherry belle plants. Six replicate plants were assessed per treatment. Bars represent SEM. In traits, where the interaction of the two factors (light intensity, TiO_2_ nanoparticle level) was significant, different letters indicate significant differences. The difference in the y-axis scale among panels ought to be noted. FW, fresh weight.

Increasing cultivation light intensity above 150 μmol m^−2^ s^−1^ PPFD tended to increase leaf total soluble carbohydrates content, with this effect being only significant at 600 µmol m^−2^ s^−1^ PPFD (Fig. 5A). At 300, and 600 µmol m^−2^ s^−1^ PPFD, the high TiO_2_ nanoparticle level (100 μmol L^−1^) stimulated leaf total soluble carbohydrates content.

Increasing cultivation light intensity enhanced tuber total soluble carbohydrates content (Fig. 5B). At 75, 150, and 300 µmol m^−2^ s^−1^ PPFD, TiO_2_ nanoparticle spray generally stimulated tuber total soluble carbohydrates content.

### TiO_2_ nanoparticle application up-regulated photosynthetic functionality under low cultivation light intensity, while down-regulated photosynthetic functionality under high cultivation light intensity

**Figure 6:**
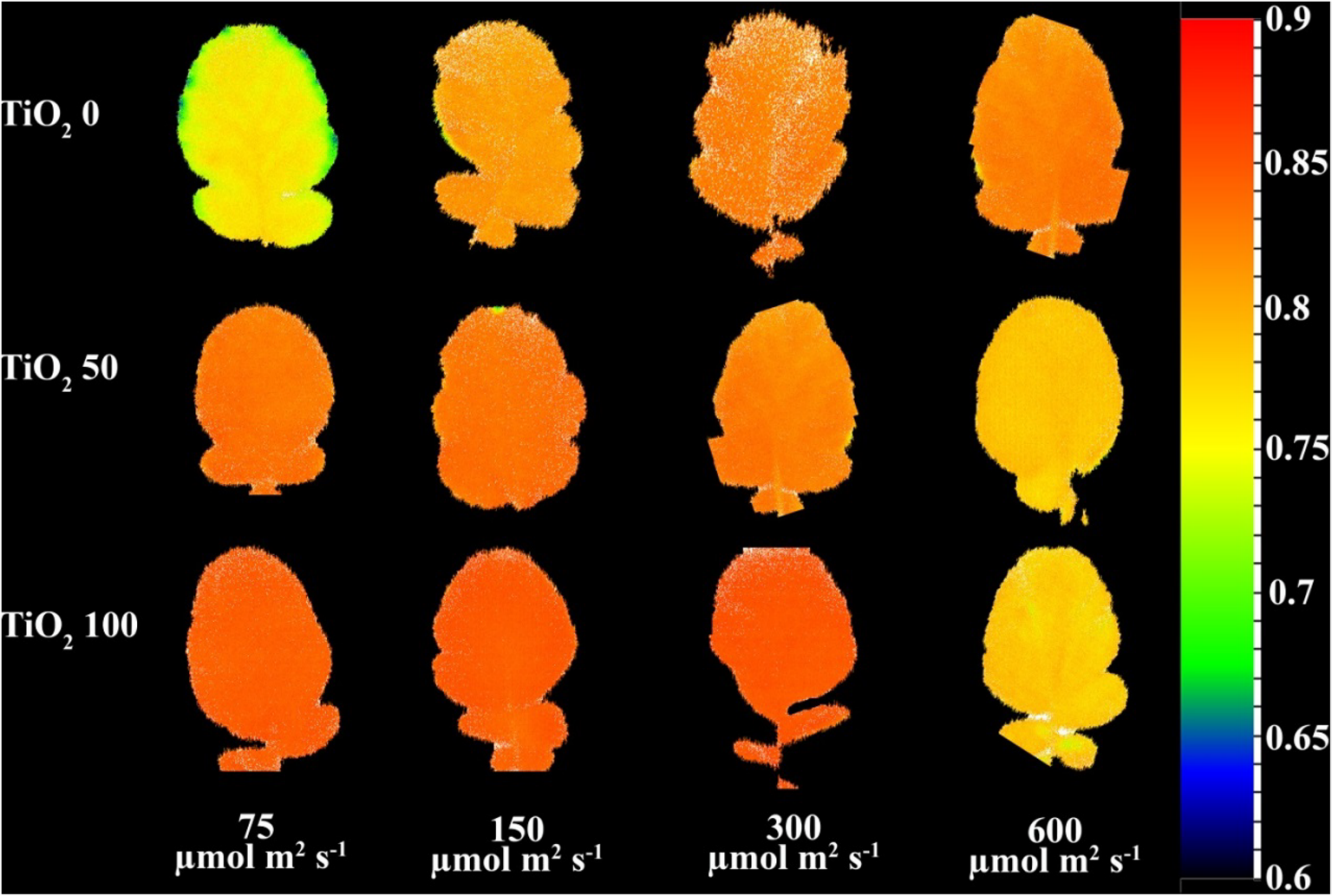
Representative image of the maximal quantum yield of PSII photochemistry (F_v_/F_m_) (equation and explanation in Table 3) of radish cv. Cherry belle plants cultivated under different light intensities (75, 150, 300 and 600 µmol m^−2^ s^−1^) and sprayed (once a week, and three times in total) with titanium dioxide (TiO_2_) nanoparticles at different concentrations (0, 50 and 100 µmol L^−1^).

At the lowest cultivation intensity (75 µmol m^−2^ s^−1^ PPFD), the lowest F_v_/F_m_ was noted (Fig. 6). At the highest cultivation intensity (600 µmol m^−2^ s^−1^ PPFD), intermediate F_v_/F_m_ values were apparent, with the highest ones being noted at 150, and 300 µmol m^−2^ s^−1^ PPFD.

At 75, and 150 µmol m^−2^ s^−1^ PPFD, TiO_2_ nanoparticle spray stimulated F_v_/F_m_ (Fig. 6). At 300 µmol m^−2^ s^−1^ PPFD, the high TiO_2_ nanoparticle level (100 μmol L^−1^) promoted F_v_/F_m_. At 600 µmol m^−2^ s^−1^ PPFD, TiO_2_ nanoparticle spray decreased F_v_/F_m_.

**Figure 7:**
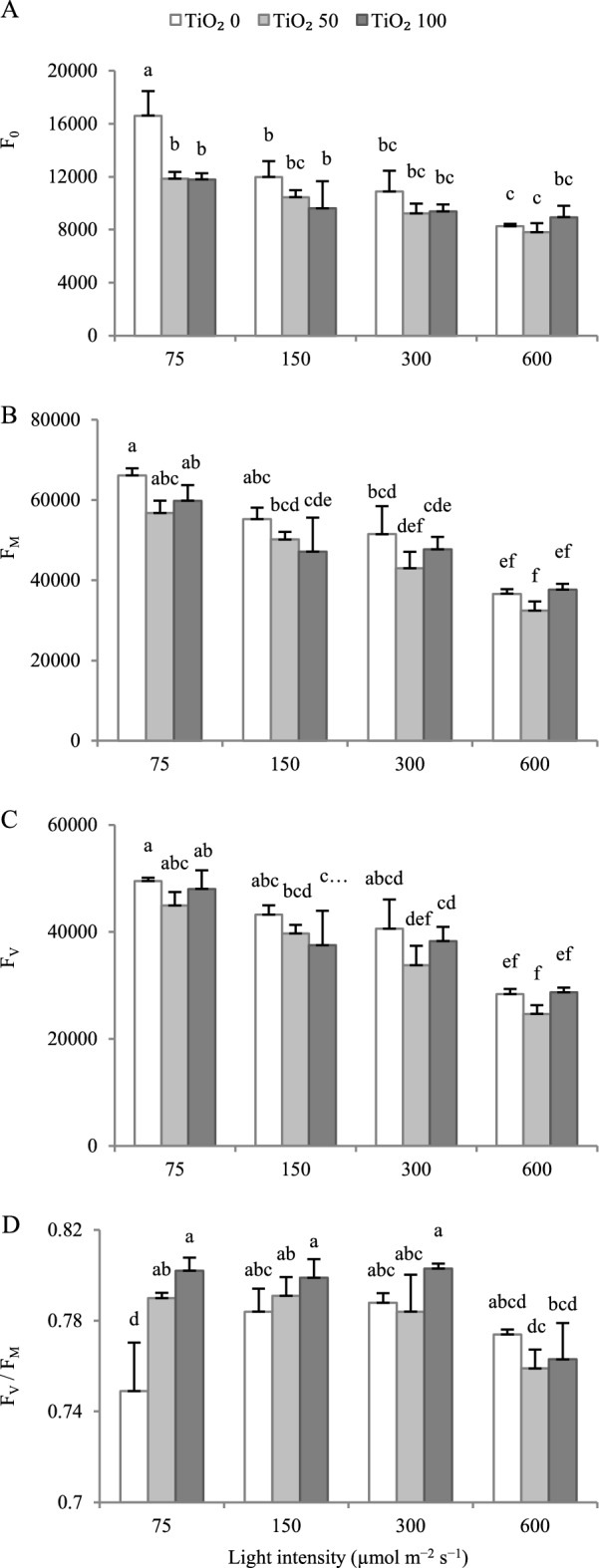
Effect of cultivation light intensity (75, 150, 300 and 600 µmol m^−2^ s^−1^) and spray treatment (once a week, and three times in total) with titanium dioxide (TiO_2_) nanoparticles (open, grey, and dark grey for 0, 50 and 100 µmol L^−1^, respectively) on minimum fluorescence when all PSII reaction centers are open (F_0_; **A**), maximum fluorescence when all PSII reaction centers are closed (F_m_; **B**), variable fluorescence of the dark-adapted sample (F_v_; **C**) and maximal quantum yield of PSII photochemistry (F_v_/F_m_; **D**) (equations and explanations in Table 3) of radish cv. Cherry belle plants. Nine replicate plants were assessed per treatment. Bars represent SEM. In traits, where the interaction of the two factors (light intensity, TiO_2_ nanoparticle level) was significant, different letters indicate significant differences.

Increasing the cultivation light intensity generally decreased the minimum fluorescence when all PSII reaction centers are open (F_0_; Fig. 7A). At 75 µmol m^−2^ s^−1^ PPFD, TiO_2_ nanoparticle spray decreased F_0_.

Increasing the cultivation light intensity generally decreased the maximum fluorescence when all PSII reaction centers are closed (F_m_; Fig. 7B) and the variable fluorescence of the dark-adapted sample (F_v_; Fig. 7C). Within each light intensity regime, TiO_2_ nanoparticle spray did not have any effect on F_m_ and F_v_.

F_v_/F_m_ was higher at 150 and 300 µmol m^−2^ s^−1^ PPFD, as compared to 75 µmol m^−2^ s^−1^ PPFD (Fig. 7D). TiO_2_ nanoparticle spray increased F_v_/F_m_ at 75 µmol m^−2^ s^−1^ PPFD.

**Figure 8:**
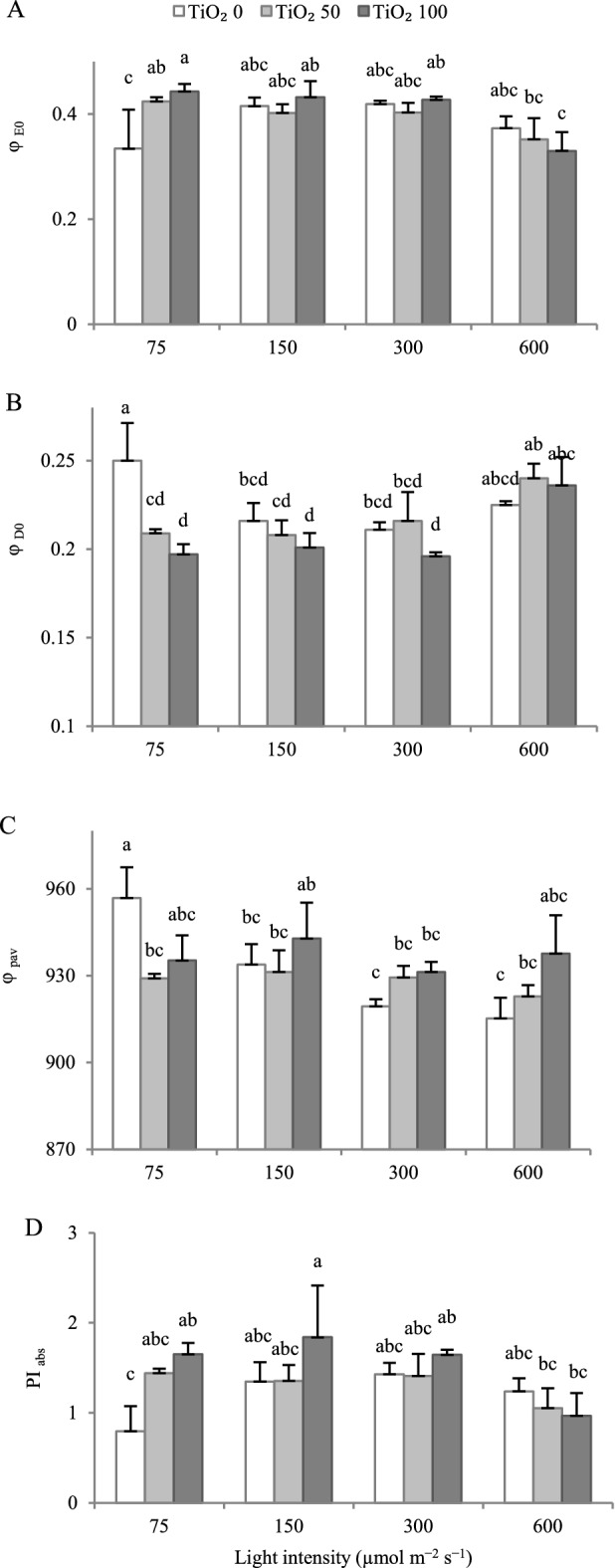
Effect of cultivation light intensity (75, 150, 300 and 600 µmol m^−2^ s^−1^) and spray treatment (once a week, and three times in total) with titanium dioxide (TiO_2_) nanoparticles (open, grey, and dark grey for 0, 50 and 100 µmol L^−1^, respectively) on quantum yield of electron transport (φ_E0_; **A**), quantum yield of energy dissipation (φ_D0_; **B**), quantum yield for primary photochemistry (φ_PAV_; **C**), performance index in light absorption basis (PI_ABS_; **D**) (equations and explanations in Table 3) of radish cv. Cherry belle plants. Nine replicate plants were assessed per treatment. Bars represent SEM. In traits, where the interaction of the two factors (light intensity, TiO_2_ nanoparticle level) was significant, different letters indicate significant differences.

Cultivation light intensity did not affect the quantum yield of electron transport (φ_E0_; Fig. 8A) and the performance index in light absorption basis (PI_ABS_; Fig. 8D). TiO_2_ nanoparticle spray increased φ_E0_ and PI_ABS_ at 75 µmol m^−2^ s^−1^ PPFD.

The quantum yield of energy dissipation (φ_D0_) was higher at 75 µmol m^−2^ s^−1^ PPFD as compared to 150 and 300 µmol m^−2^ s^−1^ PPFD (Fig. 8B). TiO_2_ nanoparticle spray decreased φ_D0_ at 75 µmol m^−2^ s^−1^ PPFD.

Increasing the cultivation intensity generally decreased the quantum yield for primary photochemistry (φ_PAV_; Fig. 8C). TiO_2_ nanoparticle spray decreased φ_PAV_ at 75 µmol m^−2^ s^−1^ PPFD.

**Figure 9:**
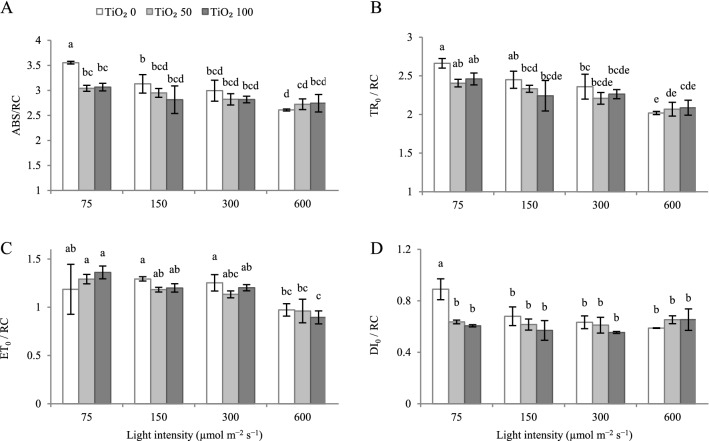
Effect of cultivation light intensity (75, 150, 300 and 600 µmol m^−2^ s^−1^) and spray treatment (once a week, and three times in total) with titanium dioxide (TiO_2_) nanoparticles (open, grey, and dark grey for 0, 50 and 100 µmol L^−1^, respectively) on specific energy fluxes per reaction center for energy absorption (ABS/RC; **A**), trapped energy flux per reaction center (TR_0_/RC; **B**), electron transport flux per reaction center (ET_0_/RC; **C**), dissipated energy flux (DI_0_/RC; **D**) (equations and explanations in Table 3) of radish cv. Cherry belle plants. Nine replicate plants were assessed per treatment. Bars represent SEM. In traits, where the interaction of the two factors (light intensity, TiO_2_ nanoparticle level) was significant, different letters indicate significant differences.

Increasing cultivation light intensity generally decreased the specific energy fluxes per reaction center for energy absorption (ABS/RC; Fig. 9A), and the trapped energy flux per reaction center (TR_0_/RC; Fig. 9B). TiO_2_ nanoparticle spray decreased ABS/RC at 75 µmol m^−2^ s^−1^ PPFD (Fig. 9A).

The electron transport flux per reaction center (ET_0_/RC) was lower at 600 µmol m^−2^ s^−1^ PPFD as compared to lower intensities (Fig. 9C). TiO_2_ nanoparticle spray did not affect ET_0_/RC.

**Figure 10:**
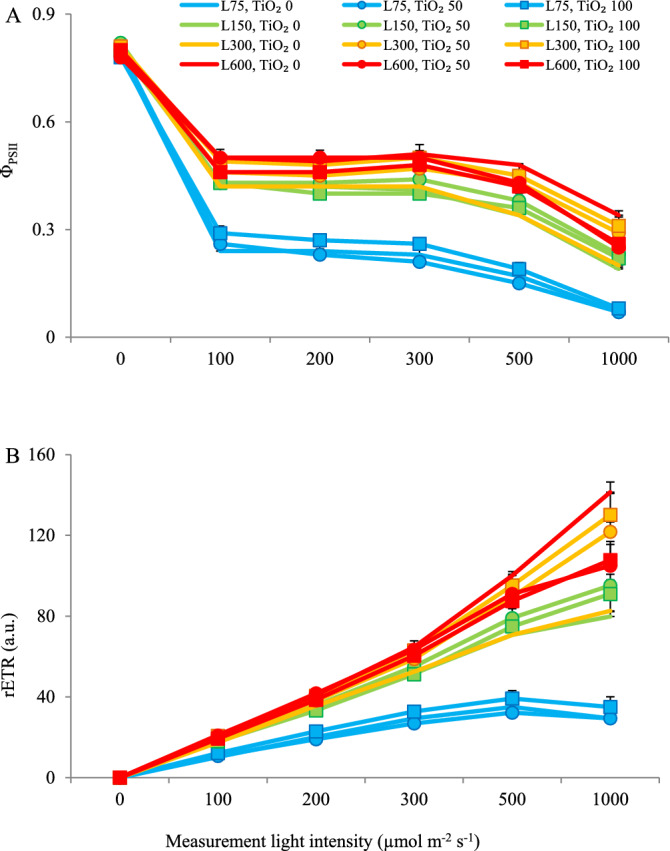
Rapid light curve of effective quantum yield of PSII (Φ_PSII_, **A**) and electron transport rate (rETR, **B**) (equations and explanations in Table 3) as a function of measurement light intensity in radish cv. Cherry belle plants cultivated under different light intensities (75, 150, 300 and 600 µmol m^−2^ s^−1^) and sprayed (once a week, and three times in total) with titanium dioxide (TiO_2_) nanoparticles at different concentrations (0, 50 and 100 µmol L^−1^). Nine replicate plants were assessed per treatment. Bars represent SEM.

The dissipated energy flux (DI_0_/RC) was higher at 75 µmol m^−2^ s^−1^ PPFD as compared to higher light intensities (Fig. 9D). TiO_2_ nanoparticle spray decreased DI_0_/RC at 75 µmol m^−2^ s^−1^ PPFD (Fig. 9D). At 0 µmol m^−2^ s^−1^ PPDF measuring light intensity, ΦPSII was not affected by cultivation light intensity (Fig. 10A). As measuring light intensity increased, ΦPSII decreased. Leaves cultivated under higher light intensities generally sustained higher ΦPSII values across measurement light levels (100−1000 μmol m^−2^ s^−1^ PPFD). At 75 µmol m^−2^ s^−1^ PPFD during cultivation, the high TiO2 nanoparticle level (100 μmol L−1) tended to sustain higher ΦPSII values. As measurement light intensity increased, rETR also increased (Fig. 10B). Differences among cultivation light intensities were magnified, as measurement light intensity increased. The lower rETR was noted in leaves cultivated under 75 µmol m−2 s−1 PPFD.

### Increasing cultivation light intensity and applying TiO_2_ nanoparticles generally stimulate non-photochemical quenching

**Figure 11:**
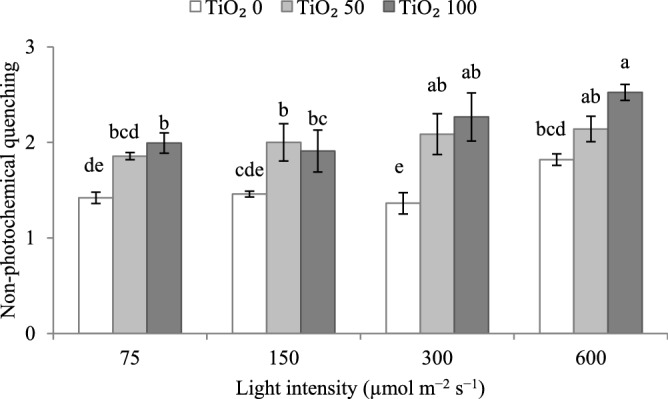
Effect of cultivation light intensity (75, 150, 300 and 600 µmol m^−2^ s^−1^) and spray treatment (once a week, and three times in total) with titanium dioxide (TiO_2_) nanoparticles (open, grey, and dark grey for 0, 50 and 100 µmol L^−1^, respectively) on non-photochemical quenching (equation and explanation in Table 3) of radish cv. Cherry belle plants. Nine replicate plants were assessed per treatment. Bars represent SEM. The interaction of the two factors (light intensity, TiO_2_ nanoparticle level) was significant, and different letters indicate significant differences.

The non-photochemical quenching (NPQ) was higher at 600 µmol m^−2^ s^−1^ PPFD as compared to lower light intensities (Fig. 11). TiO_2_ nanoparticle spray generally increased NPQ across cultivation light intensities. This effect was comparable at different cultivation light intensities.

## Discussion

In this study, the optimal concentration (50, and 100 μmol L^−1^) of TiO_2_ nanoparticles foliar application for stimulating photosynthetic efficiency, and eventually yield was evaluated in radish. There are some previous indications highlighting more effectiveness of the foliar application of TiO_2_ nanoparticles on crop growth, photosynthesis, and yield. For instance, the application of TiO_2_ nanoparticles (average diameter 25 nm, in the form of anatase, zeta potentials ˗22.5 mV) in aerosol format was more effective compared to TiO_2_ nanoparticles as a soil amendment for increasing photosynthesis and lycopene content^35^. The dependence of the respective promotive effect on cultivation light intensity (75, 150, 300 and 600 μmol m^−2^ s^−1^ PPFD) was also investigated in the present study.

Increasing cultivation light intensity resulted in a larger plant leaf area (Table 1; see also Fig. 2), and in this way improved light capture^36^. The plant leaf area increase was most prominent when light intensity increased from 75 to 150 μmol m^−2^ s^−1^ PPFD, while a further increase in light intensity led to a more moderate (plant leaf area) increase. In the former case (from 75 to 150 μmol m^−2^ s^−1^ PPFD), the increase in plant leaf area was mostly due to larger individual leaf area, whereas in the latter case (≥ 300 μmol m^−2^ s^−1^ PPFD), it was exclusively due to the larger number of leaves (thus enhanced leaf initiation). Therefore, the initial light level (rather than the percentage of increase) strongly determines both the promoting effect of increasing light intensity on plant leaf area, and the underlying process by which this increase is realized (i.e., individual leaf area and/ or the number of leaves).

When cultivation light intensity did not exceed 300 μmol m^−2^ s^−1^ PPFD, TiO_2_ nanoparticle spray also stimulated plant leaf area (Table 1; see also Fig. 2). The TiO_2_ nanoparticle spray-induced increase in plant leaf area was due to increased individual leaf area. The TiO_2_ nanoparticle spray-induced increase in plant leaf area was more prominent as cultivation light intensity was lower. Therefore, applying TiO_2_ nanoparticles provides an additional advantage in plants cultivated at low light levels.

Increasing cultivation light intensity resulted in larger plant biomass (Table 1; see also Fig. 2). The applied light levels (75–600 m^−2^ s^−1^ PPFD) were thus below the light saturation point of the crop^12^. The light intensity-induced increase in plant biomass was more prominent as the initial light intensity was lower. Across cultivation light intensities, TiO_2_ nanoparticle spray stimulated plant biomass (Table 1; see also Fig. 2). The TiO_2_ nanoparticle application-induced increase in plant biomass was also more prominent as the cultivation light intensity was lower. Both TiO_2_ nanoparticle levels (50, and 100 μmol L^−1^) were equally effective in stimulating plant biomass.

More plant mass was allocated to the leaves (i.e., higher LMR), as cultivation light intensity was lower (Table 1; Fig. 3). Thus, plants favored biomass partitioning to the light-intercepting organ (leaves) to amplify carbon gain under low light level conditions, whereas this partitioning gradually shifted to the tuber, which is evidently affiliated with the supply of other resources (i.e., water and nutrients), under higher light level circumstances^12^. Fine-tuning of leaf thickness (SLA) by regulating the light capture surface was another important aspect of adaptation to the light level in radish. Leaves were consistently thinner (i.e., higher SLA) as the light level decreased (Table 1). Increasing SLA stimulates the amount of light which is intercepted, and in this way maximizes carbon assimilation. This adaptation in leaf morphology is common in low-light situations, and has been reported in several taxa^12^.

Throughout the distribution chain of edible greens, the intensity of leaf greenness is an index of quality^5^. At inadequate leaf chlorophyll level, photosynthetic efficiency is also impeded^23^. Carotenoids are the main antioxidant metabolites, contributing to plant defense, and exhibiting health-promoting properties when consumed^5^. Increasing cultivation light intensity up to 300 μmol m^−2^ s^−1^ PPFD improved both leaf chlorophyll and carotenoid contents, while a further increase (600 μmol m^−2^ s^−1^ PPFD) exerted an adverse effect (Fig. 4). At 75, 150, and 600 μmol m^−2^ s^−1^ PPFD, TiO_2_ nanoparticle spray promoted leaf chlorophyll content (Fig. 4A). At 75, and 600 μmol m^−2^ s^−1^ PPFD, TiO_2_ nanoparticle spray also enhanced leaf carotenoid content (Fig. 4B). In either case, the optimum TiO_2_ nanoparticle concentration depended on light level. Therefore, applying TiO_2_ nanoparticles improves both leaf chlorophyll and carotenoid contents, especially under extreme light environments (i.e., low or very high).

Increasing cultivation light intensity generally stimulated the content of total soluble carbohydrates (primary energy reserves) in both leaves and tuber, with this effect being more prominent in the tuber (Fig. 5). TiO_2_ nanoparticle spray further improved both leaf and tuber total soluble carbohydrates contents, with this effect being more pronounced in the leaf (Fig. 5). The level of primary energy reserves has been earlier associated with prolonged postharvest longevity^22,37^. For instance, an increased content of soluble carbohydrates may support maintenance needs (by sustaining ATP synthesis), and protect membrane integrity (by scavenging reactive oxygen species)^22,37^.

Growth environment determines the light energy absorbance by the PSII apparatus, and this is readily reflected by chlorophyll fluorescence analysis^38^. As growth PPFD increased, a decreasing trend was noted in the F_0_, F_m_, and F_v_ (Fig. 7A–C). These parameters (F_0_, F_m_, and F_v_) were generally not affected by TiO_2_ nanoparticle spray, except for a decrease in F_0_ at 75 µmol m^−2^ s^−1^ PPFD. The decrease in F_0_ may be associated with a reduction in plastoquinone electron receptors and incomplete oxidation due to retardation, which results in electron transfer chain postponement in PSII. Alternatively, F_0_ may be decreased by the separation of light-harvesting Chl a/b protein complexes in PSII^39^. The reduction in F_m_ is probably related to the deactivation of proteins in chlorophyll structure.

Higher values of F_v_/F_m_ [(F_m_ – F_0_)/F_m_; Table 3] reflect a better light use efficiency^39^. Under stress, both the rising trend of F_0_ and the declining trend of F_m_ typically underlie the decrease in F_v_/F_m_^15,25,40^. This decline in F_v_/F_m_ denotes a stress-induced damage in the structure or function of PSII. As growth PPFD increased up to 300 µmol m^−2^ s^−1^ PPFD, F_v_/F_m_ also increased (Fig. 7D; see also Fig. 6). A further increase in growth PPFD to 600 µmol m^−2^ s^−1^ led to a decrease in F_v_/F_m_. Therefore, the optimum growth light intensity for light use efficiency was clearly the 300 µmol m^−2^ s^−1^ PPFD. In lettuce, the optimum growth light intensity was 600 µmol m^−2^ s^−1^ PPFD^16,17,41–44^, while for *Aloe vera* L. it was 50% of sunlight as compared to 75 or 100%^44^. F_v_/F_m_ was generally not affected by TiO_2_ nanoparticle spray, except for an increase noted at 75 µmol m^−2^ s^−1^ PPFD. Therefore, TiO_2_ nanoparticle spray improved light use efficiency at the lowest growth light intensity.

PI_ABS_ is an index of the quantum yield of absorbed photons, and is highly sensitive to changes in the concentration of reaction centers, initial photochemistry, and electron transport^14,45^. Exposure to non-optimal PPFD leads to alterations in absorption and energy trapping, thereby adversely influencing PI_ABS_^17^. Similarly to F_v_/F_m_, PI_ABS_ increased as growth PPFD increased up to 300 µmol m^−2^ s^−1^ PPFD, and then (600 µmol m^−2^ s^−1^ PPFD) slightly decreased (Fig. 8D). Likewise to F_v_/F_m_, TiO_2_ nanoparticle spray increased PI_ABS_ at 75 µmol m^−2^ s^−1^ PPFD. A decrease in PI_ABS_ may be due to the higher light energy absorption, dissipated energy flux, and lower electron transport per reaction center (expressed by ABS/RC, DI_0_/RC, and ET_0_/RC, respectively)^46^.

ABS/RC flux is divided into TR_0_/RC, ET_0_/RC, and DI_0_/RC, while the share of ABS is converted to DI and TR. As growth PPFD increased, ABS/RC tended to decrease (Fig. 9A). ABS/RC was generally not affected by TiO_2_ nanoparticle spray, except for a decrease apparent at 75 µmol m^−2^ s^−1^ PPFD. The decreasing trend in ABS/RC indicates the more efficient function of the electron transport system, which decreases the excitation force on each reaction center and directs that energy mostly toward photosystem I.

At 600 µmol m^−2^ s^−1^ PPFD, ET_0_/RC was lower (Fig. 9C), indicating an adverse effect in the rate of electron transport flux on PSII reaction centers. Increasing cultivation light intensity generally decreased TR_0_/RC (Fig. 9B). At 75 µmol m^−2^ s^−1^ PPFD, DI_0_/RC was higher (Fig. 9D). These parameters (ET_0_/RC, TR_0_/RC, and DI_0_/RC) were generally not affected by TiO_2_ nanoparticle spray, except a decrease in DI_0_/RC at 75 µmol m^−2^ s^−1^ PPFD. A high DI_0_/RC is generally associated with decreased F_v_/F_m_ and an increased incidence of photoinhibition^17,47^. Since DI_0_/RC is related to the partial deactivation of PSII reaction centers, it usually increases when PSII reaction centers cannot transfer energy upstream of PSII as a consequence of damage to the thylakoid membrane^14,48^. Shabbir et al.^49^ reported that the photochemical efficiency of PSII increased by applying 90 mg L^−1^ TiO_2_ to vetiver plants. In the study by Gao et al.^50^ treatment of spinach with TiO_2_ resulted in the induction of a complex of Rubisco and Rubisco activase to accelerate the carboxylation of Rubisco and ultimately improve photosynthetic efficiency. In the study of Azmat et al.^51^, the treatment of spinach with TiO_2_ nanoparticles increased the starch content due to its photocatalytic properties.

The absorbed light energy is quenched via photochemistry, fluorescence, or heat dissipation. In a healthy photosystem, a large portion is used to perform photosynthesis (the so-called photochemical quenching), and a trivial one is released via fluorescence. The third fate is NPQ, in which the excess excitation energy of the chlorophyll in the PSII complex is harmlessly dissipated into thermal energy^52^. Therefore, NPQ signifies an effective way for dissipating excessive irradiation into heat by the photosynthetic apparatus^53^. Studying the behavior of NPQ provides information regarding the xanthophyll cycle activity and other energy-quenching pathways which are induced by the proton concentration inside thylakoids^54–56^. NPQ was higher at 600 µmol m^−2^ s^−1^ PPFD (Fig. 11). Across cultivation light intensities, TiO_2_ nanoparticle spray generally increased NPQ. Thus, it can be concluded that plants grown under the highest PPFD and/ or TiO_2_ nanoparticle spray directed more efficiently the excess excitation energy through non-radiative dissipative processes. This high energy input can be due to feedback inhibition of carbohydrate accumulation on the electron transport chain. An increase in the level of carotenoids as a result of exposure to higher PPFDs or TiO_2_ concentrations is a helpful strategy to direct the energy input toward the NPQ. In this way, the photosynthetic apparatus remains adequately protected under conditions of excessively high light energy input.

The rapid light curves illustrate the short-term photosynthetic response to rising light levels. Studying them is a proper way to gain knowledge on overall photosynthetic performance as well as the saturation characteristics of electron transport^49^. In the present study, rapid light curves of both Φ_PSII_ and rETR were assessed. As measuring light intensity increased, Φ_PSII_ decreased whereas rETR increased (Fig. 10). The decline in Φ_PSII_ denotes a limited capacity for photochemical energy usage. In rETR, both the lower increase and the development of a plateau depict the light intensity, where the photosynthetic pathway became limited. Across measurement light levels (100 − 1000 μmol m^−2^ s^−1^ PPFD), leaves cultivated under higher light intensities generally sustained higher Φ_PSII_ and rETR values (Fig. 10). At 75 µmol m^−2^ s^−1^ PPFD during cultivation, TiO_2_ nanoparticle spray (100 μmol L^−1^) tended to sustain higher Φ_PSII_ and rETR values. In accordance, it has been shown that foliar spraying of 100 mg L^−1^ of TiO_2_ nanoparticles under low light intensity increased the rate of photosynthesis and yield in cherry tomato plants^30^.

**Figure 12:**
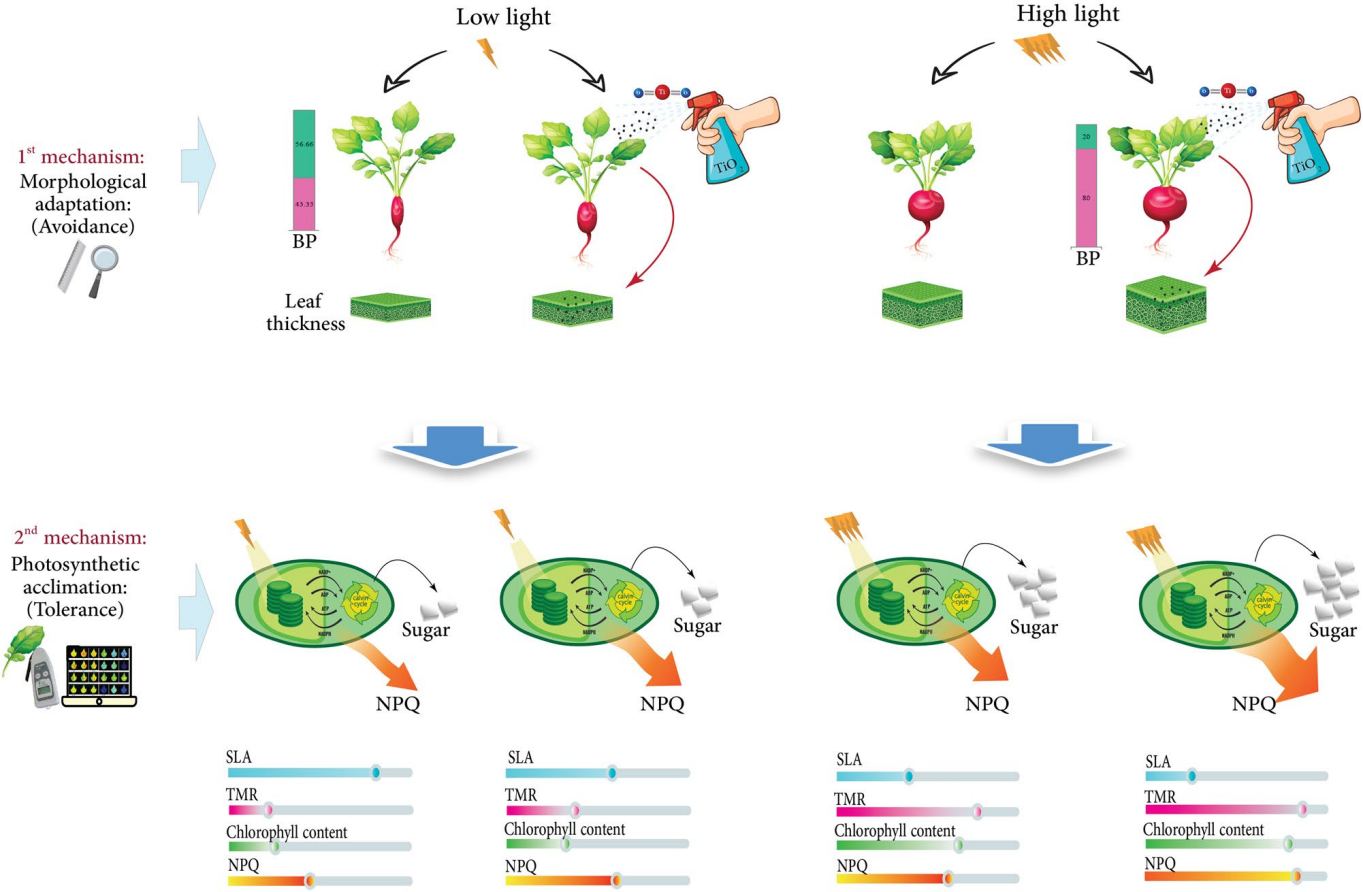
A schematic illustration on the effect of low and high cultivation light intensity and titanium dioxide (TiO_2_) nanoparticles application on radish plants. Under low light, plants partitioned less biomass towards the tubers and allocated their biomass toward leaf expansion rather than leaf thickness. Low light-exposed plants decreased NPQ and accumulated less carbohydrates. Conversely, under high light, plants partitioned most of their biomass into the tubers and produced thick leaves with limited area. They increased NPQ and produced more carbohydrates under high light condition. TiO_2_ nanoparticle application directed more biomass into the tubers under low light intensities, while increased NPQ under high light condition.

In conclusion, radish plants employed two mechanisms under contrasting lighting conditions. In the first mechanism (morphological adaptation), under low light, plants partitioned their biomass toward upper-ground parts which resulted in smaller tubers. Furthermore, plants allocated their biomass toward leaf expansion rather than leaf thickness which was indicated by the high SLA of plants under low light intensity (shade avoidance response). Conversely, plants under high light partitioned most of biomass toward underground parts which resulted in production of bigger tubers. They also produced thicker leaves with limited area to avoid capturing excessive light photons. In the second mechanism (photosynthetic acclimation), plants decreased NPQ and accumulated less carbohydrates under low light condition, while increased NPQ and produced more carbohydrates under high light condition. TiO_2_ nanoparticle application mitigate the shade avoidance response under low light intensities, while increased NPQ and reduced electron transport under high light due to higher energy perception (Fig. 12).

## Materials and methods

### Plant material and growth conditions

Seeds of radish (*R. raphanistrum*) cv. Cherry Belle were sterilized (3 min) in sodium hypochlorite solution (5%; v/v), and then rinsed (3 min) with distilled water. Seeds were then manually planted in a seedling tray (30 × 18 × 5 cm) filled with a mixture of peat and perlite (1:1, v/v). The sowing depth was kept constant, and seeds were covered with substrate immediately after sowing. At the two-leaf stage, plants were transferred to 2 L pots [diameter (top) × diameter (bottom) × height = 15 × 13 × 15 cm] containing the same mixture. To facilitate drainage, 5 ± 1 g of pebbles (particle size of 16–30 mm) had been earlier placed at the bottom of each pot. Next, 120 pots were equally distributed to four environmentally-controlled growth chambers (l × w × h = 1.5 × 1.0 × 1.3 m) for realizing treatments. A density of 20 plants per m^2^ was employed.

Twelve factors (4 light intensities × 3 TiO_2_ nanoparticle spray treatments) were applied as a factorial experiment based on a completely randomized design. Each growth chamber included a single light intensity level, which was set at 75, 150, 300 and 600 μmol m^−2^ s^−1^ PPFD for 16 h d^−1^ (06:00–22:00), respectively. In all chambers, light was provided by red (660 nm peak wavelength) and blue (450 nm peak wavelength) LED modules (Parcham Co, Tehran, Iran) with a (red to blue) ratio of 3 to 1. Light intensity was determined by a handy fluorometer (PAR-FluorPen FP 100-MAX, Photon Systems Instruments, Drásov, Czech Republic), while the spectrum was defined by a handy spectrometer (Seconic C7000, Japan).

The remaining environmental variables were identical among the four growth chambers. Day/ night air temperature was set to 26/ 16 °C, and relative air humidity was adjusted to 65–70%. Two ventilation fans (12 V, 0.90A) were installed in each growth chamber to ensure uniform air circulation. Plants were cultivated in a customized liquid culture hydroponic system, by using a modified Hoagland nutrient solution (pH ≈ 5.8; EC ≈ 1.8 dS m^−1^; detailed composition in Table 2). Growth media moisture was maintained at or near maximum water-holding capacity. To prevent salt accumulation, pots were also irrigated with distilled water twice a week (> 20% drainage).

**Table 2:** Stock solutions' ingredients employed in the present study. From each stock solution, 10 mL was added to prepare 1 L of nutrient solution.

Ingredient	Quantity for 1 L of stock solution (g)
Calcium nitrate	97
Potassium nitrate	61
Magnesium sulfate (16% MgO)	25
Mono-potassium phosphate	19
Potassium sulfate	17
Iron EDDHA (6%)	3.723
Mono-ammonium phosphate	1
Borax (11.3% B)	0.383
Zinc sulfate	0.35
Manganese sulfate	0.3
Copper sulfate	0.04
Sodium molybdate (39.6%)	0.024

Plant- and leaf-level measurements were conducted. For leaf-level measurements, sampled leaves had grown under direct light, and were fully-expanded. Replicate leaves were selected from separate plants. To minimize border effects, experimental plants were surrounded by border plants (adjacent to chamber walls) that were not sampled. Samples were collected at the onset of the light period (06:00–07:00 h). Different treatments were always assessed simultaneously. In growth and biomass allocation measurements, the time between sampling and the start of the evaluation did not exceed 15 min. In the remaining evaluations, samples were placed in vials, flash frozen in liquid nitrogen, and transferred to a freezer (− 80 °C) for storage. In all cases, sampling was conducted at the end of the cultivation period, besides the chlorophyll fluorescence evaluation, which was performed 1 d earlier. In all determinations, six replicates were assessed per treatment, besides chlorophyll fluorescence assessments, where nine replicates were evaluated.

### Preparation of TiO_2_ suspension

The methods used for the synthesis of TiO_2_ nanoparticles are Sol–gel route, fame hydrolysis, co-precipitation, impregnation and chemical vapor deposition. Three crystalline phases of titanium dioxide, are anatase (tetragonal), rutile (tetragonal), and brookite (orthorhombic), that brookite has no commercial value^11^. TiO_2_ nanoparticles in the anatase form (˃ 99% purity, APS: 10–25 nm, Color: white, Bulk density: 0.24 g/cm^3^, True density: 3.9 g/cm^3^, PH: 6–6.5) were obtained from a commercial supplier (Iranian Nanomaterial Pioneers Company, Mashhad, Iran). The shape, size and crystal structure of TiO2 nanoparticles were evaluated using SEM, TEM, XRD and FTIR. The Specific surface area (SSA) and pores were analyzed by BET (Brunauer–Emmett–Teller) instrument.

In each light intensity regime, following adaptation (3 d), TiO_2_ nanoparticles at different concentrations (0, 50, and 100 μmol L^−1^) were weekly applied (three times in total) via foliar spray. The suitable concentration range was selected based on both a comprehensive literature survey^20,28,57^, and a pre-experiment. Each time before application, the TiO_2_ nanoparticles solution was ultrasonically homogenized using a sonicator (UP100H, Hielscher Ultrasonics, Germany)^58–60^. At the time of the first application, plants were still at the two-leaf stage.

### Plant growth, morphology, and biomass allocation

The petiole (stalk) length (from the base to the leaf joint), the number of leaves, individual leaf area (one-sided surface area), and plant leaf area were first determined. For leaf area assessment, leaves were scanned (HP Scanjet G4010, Irvine, CA, USA) and then evaluated by using the Digimizer software (version 5.3.5, MedCalc Software, Ostend, Belgium)^21,61^.

Following removal of the substrate from the tuber via gentle washing, tuber volume was measured by employing a volume-displacement technique^62,63^. Tubers were suspended in a cylinder filled with water, and tuber volume was then determined by measuring the volume of displaced water.

Leaf and tuber (fresh and dry) masses were also recorded (± 0.01 g; MXX-412; Denver Instruments, Bohemia, NY, USA). For measuring dry weight, samples were placed in a forced-air drying oven for 72 h at 80 °C. By using dry mass, specific leaf area (SLA; leaf area/leaf mass), leaf mass ratio (LMR; leaf mass/plant mass), and tuber mass ratio (TMR; tuber mass/plant mass) were calculated.

Prior to the destructive measurements, plant images were obtained by using a digital camera (Canon EOS M2; Canon Inc., Tokyo, Japan). The plants were manually moved to the image capture station. The imaging station included top and side lighting units (fluorescent tubes; Pars Shahab Lamp Co., Tehran, Iran). The camera-to-plant distance was maintained constant. One image (RGB) was obtained per plant.

### Leaf chlorophyll and carotenoid content

Samples (0.1 g) were homogenized with the addition of 10 mL of 100% acetone. The extract was then centrifuged (14,000 g for 15 min), and the supernatant was collected. Since chlorophyll is light sensitive, the extraction took place in a dark room^58,59^. The obtained extract was subjected to reading on a spectrophotometer (Optizen pop, Mecasys Co. Ltd. Daejeon, Korea). Total chlorophyll and carotenoid contents were calculated according to Lichtenthaler and Wellburn^64^.

### Leaf total soluble carbohydrates content

Colorimetric quantification of leaf total soluble carbohydrates (i.e., reducing and non-reducing sugars) content was performed^65^. Shortly before analysis (< 30 min), the anthrone reagent was prepared under darkness by dissolving 0.1 g of anthrone (0.2%) in 100 mL of concentrated sulfuric acid (98%). Sample (0.1 g) and anthrone reagent (1 mL) were loaded in tubes, which were placed in a water bath (90 °C for 15 min), cooled (0 °C for 5 min), and vortexed (1 min). Before reading, a heating step (20 min) to room temperature (25 °C) was performed. The absorbance was measured at 620 nm with a spectrophotometer (Optizen pop, Mecasys Co. Ltd. Daejeon, Korea). A standard curve based on a series of known glucose concentrations was prepared.

### Maximum quantum yield of photosystem II (PSII)

As a sensitive indicator of photosynthetic performance, dark-adapted values of the maximum quantum yield of PSII (F_v_/F_m_; equation and explanation in Table 3) were non-invasively recorded in leaves of each treatment^22,37,63^. Measurements were performed by using a portable imaging fluorometer (Handy FluorCam FC 1000-H, Photon Systems Instruments, Drásov, Czech Republic). Before taking measurements, samples were dark-adapted (≥ 20 min). Then, F_v_/F_m_ was evaluated by a custom-made protocol^21,23^, where leaves were exposed to short flashes followed by long saturated light pulses (3900 µmol m^−2^ s^−1^ PPFD) to cause a reduction in the primary quinone acceptor of PSII.

**Table 3:** Abbreviations, definitions and formulas of the chlorophyll fluorescence parameters assessed in the current study.

Category	Abbreviation	Definition	Equation
Basic parameters	F_0_	Minimum fluorescence, when all PSII reaction centers are open (O-step of OJIP transient)	F_50µs_
	F_J_	Fluorescence intensity at the J-step (2 ms) of OJIP	F_2ms_
	F_I_	Fluorescence intensity at the I-step (30 ms) of OJIP	F_30ms_
Fluorescence parameters	F_m_	Maximum fluorescence, when all PSII reaction centers are closed (P-step of OJIP transient)	F_1s_ = F_p_
	F_v_	Variable fluorescence of the dark-adapted sample	F_m_ – F_0_
	V_J_	Relative variable fluorescence at time 2 ms (J-step) after start of actinic light pulse	(F_J_ – F_0_)/(F_m_ – F_0_)
	V_I_	Relative variable fluorescence at time 30 ms (I-step) after start of actinic light pulse	(F_30ms_ – F_0_)/(F_m_ – F_0_)
	F_v_/F_m_	Maximal quantum yield of PSII photochemistry	1 – (F_0_/F_m_) = (F_m_ – F_0_)/F_m_ = φ_P0_ = TR_0_/ABS
Quantum yields and efficiencies/probabilities	φ_E0_	The quantum yield of electron transport	[1– ( F_0_/ F_m_)](1 – V_J_) = ET_0_/ABS
	φ_D0_	Quantum yield of energy dissipation	F_0_/F_m_
	φ_PAV_	Average (from time 0 to t_FM_) quantum yield for primary photochemistry	φ_P0_ (1 − V_J_) = φ_P0_ (S_M_/t_FM_)
Specific energy fluxes (per Q_A_ reducing PSII reaction center)	ABS/RC	The specific energy fluxes per reaction center for energy absorption	M_0_ (1/V_J_)(1/φ_P0_)
	TR_0_/RC	Trapped energy flux (leading to Q_A_ reduction) per reaction center	M_0_ (1/V_J_)
	ET_0_/RC	Electron transport flux (further than Q_A_^−^) per reaction center	M_0_ (1/V_J_)(1 − V_J_)
	DI_0_/RC	Dissipated energy flux	(ABS/RC) − (TR_0_/RC)
Performance indices (products of terms expressing partial potentials at steps of energy bifurcations)	PI_ABS_	Performance index for the photochemical activity	[(γRC/1 − γRC)( φ_P0_ /1 − φ_P0_)(ψ_E0_ /1 − ψ_E0_)]
	NPQ	Non-photochemical quenching	(F_m_/F_m_´) – 1
Rapid light curve as a function of PPFD	Φ_PSII_	Effective quantum yield of PSII	∆F/F_m_´ = (F_m_´ – F)/ F_m_´
	rETR	Relative electron transport rate	Φ_PSII_ × PPFD × 0.5 × 0.84

### Polyphasic chlorophyll fluorescence transient (O–J–I–P) evaluation

A polyphasic chlorophyll fluorescence induction curve (O–J–I–P-transient) was obtained in the attached leaves of each treatment. By employing this test, the shape changes of the O–J–I–P transient are quantitatively translated to a set of parameters (see Table 3), which relate to the in vivo adaptive behavior of the photosynthetic apparatus (especially PSII) to the growth environment^25,26^. Measurements were conducted by using a PAR- FluorPen FP 100-MAX (Photon Systems Instruments, Drásov, Czech Republic) following dark adaptation (≥ 20 min). The employed light intensity (3900 μmol m^−2^ s^−1^ PPFD) was sufficient to generate maximal fluorescence in all treatments.

Following dark adaptation, leaves exhibit a polyphasic chlorophyll fluorescence rise during the first second of illumination. The fluorescence transient, plotted on a logarithmic time scale, typically includes the following phases: O to J, J to I, and I to P. F_0_ was measured at 50 μs, fluorescence intensity at J-step was assessed at 2 ms, and fluorescence intensity at I-step was evaluated at 30 ms^39,66^.

### Non-photochemical quenching

Another set of dark-adapted attached leaves were used to determine non-photochemical quenching (NPQ; equation and description in Table 3) by using a PAR-FluorPen FP 100-MAX (Photon Systems Instruments, Drásov, Czech Republic). Calculations were performed by using the FluorPen software (v. 7; Photon Systems Instruments, Drásov, Czech Republic).

### Rapid light curve of effective quantum yield of PSII and electron transport rate

The rapid light curve of effective quantum yield of PSII (Φ_PSII_; equation and description in Table 3) was determined with a handy fluorometer (PAR-FluorPen FP 100-MAX, Photon Systems Instruments, Drásov, Czech Republic). The dark-adapted leaves were exposed to a gradual increase of actinic blue light illumination in six steps (0, 100, 200, 300, 500 and 1000 μmol m^−2^ s^−1^ PPFD). Each illumination step was separated by 10 s intervals. Illuminating the saturating flash required 0.8 s. Data were analyzed by using the FluorPen software (v. 7; Photon Systems Instruments, Drásov, Czech Republic). Relative electron transport rate (rETR; equation and description in Table 3) was also calculated^54^.

### Statistical analysis

Data were subjected to analysis of variance using SAS software (v. 9.4, SAS Institute Inc., Cary, NC, USA). Data were firstly tested for normality (Shapiro–Wilk test) and homogeneity of variances (Levene’s test). A split-plot design was employed, with cultivation light intensity (75, 150, 300 and 600 μmol m^−2^ s^−1^ PPFD) as the main factor, and TiO_2_ nanoparticles concentration (0, 50, and 100 μmol L^−1^) as the split factor. Treatment effects were tested at a 5% probability level and mean separation was carried out using the least significant differences based on Duncan's multiple range test (*P* ≤ 0.05).

## Data Availability

The main data are contained within the article. Data are available upon request from the corresponding author.

## Abbreviations

ABS/RC: The specific energy fluxes per reaction center for energy absorption
DI_0_/RC: The dissipated energy flux
ET_0_/RC: The electron transport flux per reaction center
F_m_: The maximum fluorescence when all PSII reaction centers are closed
F_v_: The variable fluorescence of the dark-adapted sample
F_v_/F_m_: Ratio of variable to maximum fluorescence
FW: Fresh weight
F_0_: The minimum fluorescence when all PSII reaction centers are open
LMR: Leaf mass ratio
NPQ: Non-photochemical quenching
PI_ABS_: The performance index in light absorption basis
PPFD: Photosynthetic photon flux density
PSII: Photosystem II
rETR: Relative electron transport rate
SLA: Specific leaf area
TEM: Transmission electron microscopy
TiO_2_: Titanium dioxide
TMR: Tuber mass ratio
TR_0_/RC: The trapped energy flux per reaction center
XRD: X-ray diffraction
∆F/F_m′_: Effective quantum yield of PSII
φ_D0_: The quantum yield of energy dissipation
Φ_PSII_: Effective quantum yield of PSII
φ_E0_: The quantum yield of electron transport
φ_PAV_: The quantum yield for primary photochemistry
